# Effects of Body Mass Index, Waist Circumference, Waist-to-Height Ratio and Their Changes on Risks of Dyslipidemia among Chinese Adults: The Guizhou Population Health Cohort Study

**DOI:** 10.3390/ijerph19010341

**Published:** 2021-12-29

**Authors:** Li Cao, Jie Zhou, Yun Chen, Yanli Wu, Yiying Wang, Tao Liu, Chaowei Fu

**Affiliations:** 1 Key Laboratory of Public Health Safety & NHC Key Laboratory of Health Technology Assessment, School of Public Health, Fudan University, Shanghai 200032, China; 21211020001@m.fudan.edu.cn (L.C.); 18211020001@fudan.edu.cn (Y.C.); 2 Guizhou Center for Disease Control and Prevention, Guiyang 550004, China; zhoujie19872014@163.com (J.Z.); wuyanli871009@163.com (Y.W.); wyy123789123789@163.com (Y.W.)

**Keywords:** body mass index, waist circumference, waist-to-height ratio, dyslipidemia

## Abstract

This study aimed to assess the effects of different anthropometric indices and their changes on the risk of incident dyslipidemia among the Chinese population. From the Guizhou population health cohort study, 2989 Chinese adults without dyslipidemia at baseline were followed up. Anthropometric parameters including waist circumference (WC), body mass index (BMI), waist-to-height ratio (WHtR), and their changes in the latter two indices, and serum lipids were tested after at least 8 h fasting. Hazard ratio (HR), adjusted hazard ratio (aHR), and 95% confidential interval (CI) were calculated to estimate the association between anthropometric parameters and dyslipidemia risk using multivariate Cox regression. A total of 2089 (69.98%) new dyslipidemia cases were identified over an average follow-up of 7.0 years. Baseline BMI (aHR = 1.12, 95%CI 1.01, 1.23) and WHtR (aHR = 1.06, 95%CI 1.00, 1.13) were positively associated with higher risks of incident dyslipidemia but not WC. Each 5.0 kg/m^2^ increment of BMI or 0.05-unit increment of WHtR was significantly associated with 43% or 25% increased risk of incident dyslipidemia, respectively. The aHRs (95%CI) of incident dyslipidemia for subjects maintaining or developing general obesity were 2.19 (1.53, 3.12) or 1.46 (1.22, 1.75), and 1.54 (1.23, 1.82) or 1.30 (1.06, 1.60) for subjects maintaining or developing abdominal obesity, respectively. Linear trends for aHRs of BMI, WHtR change, and BMI change were observed (*p* for trend: 0.021, <0.001, <0.001, respectively). BMI, WHtR, and their changes were closely associated with the incidence of dyslipidemia for Chinese adults. Loss in BMI and WHtR had protective effects on incident dyslipidemia, whereas gain of BMI or WHtR increased the dyslipidemia risk. Interventions to control or reduce BMI and WHtR to the normal range are important for the early prevention of dyslipidemia, especially for participants aged 40 years or above, male participants, and urban residents with poor control of obesity.

## 1. Introduction

Dyslipidemia, a metabolic disease characterized by a high level of fats in blood, which can build up and clog the blood vessels in heart [1], is an established and modifiable risk factor for cardiovascular disease (CVD) [2]. It is also the leading cause of atherosclerosis, which is inextricably linked with the development of CVD [3]. CVD has been the leading cause of death worldwide [4] and accounted for 45.50% and 43.16% of all deaths in rural and urban areas of China in 2016, respectively [5]. Of note, weak awareness and management of dyslipidemia were reported among Chinese adults [6]. In recent years, the prevalence of dyslipidemia among the general population of China has continuously increased [7], although previous studies indicated that early screening and effective control of dyslipidemia may help to reduce the morbidity and mortality due to CVD [8,9]. Therefore, it is of vital importance to identify the potential influencing factors and early predictors of dyslipidemia.

The occurrence and development of dyslipidemia are affected by various factors such as age, family history, unhealthy lifestyle, and other metabolic disorder statuses [1,7]. It is believed that obesity is intimately correlated with dyslipidemia, which is mainly driven by the effects of insulin resistance and proinflammatory adipokines [10]. Body mass index (BMI) measured by a combination of weight and height is widely used in assessing the obesity status, but it cannot describe the distribution of abdominal adipose tissue like the waist-to-height ratio (WHtR) or waist circumference (WC) does [11]. Cross-effects among indicators also need consideration. For example, abdominal fat accumulation leads to weight gain, subsequently contributing to an increase in BMI [12]. Therefore, it is necessary to explore and compare the predictive power of different obesity indicators on the risk of dyslipidemia simultaneously. Accumulating studies had been conducted to explore associations between different anthropometric obesity indices like BMI [13,14,15], WC [14,15,16], WHtR [16,17,18], and the risk of dyslipidemia. However, most of them were cross-sectional studies [13,15,16,17,18], which could not make causal association between obesity status and the dyslipidemia, and those findings were controversial. Changes in anthropometric indices were better for assessing the effect of excess fat [19], and some longitudinal studies assessed possible impacts of long-term changes in anthropometric indices on the risk of incident dyslipidemia [2,20,21].

To the best of our knowledge, longitudinal studies systematically evaluating the risk of incident dyslipidemia based on different anthropometric parameters and their changes among Chinese population were not reported so far. Furthermore, different effects over sociodemographic factors should be well explored to help to target populations at risk. Therefore, based on the Guizhou Population Health Cohort, we aimed to examine effects of BMI, WC, WHtR, BMI changes, and WHtR changes on the risk of incident dyslipidemia and possible interactions between different anthropometric obesity indices and sociodemographic factors in southwest China, which may benefit the prevention and control of dyslipidemia.

## 2. Materials and Methods

### 2.1. Study Population and Procedure

**Figure 1:**
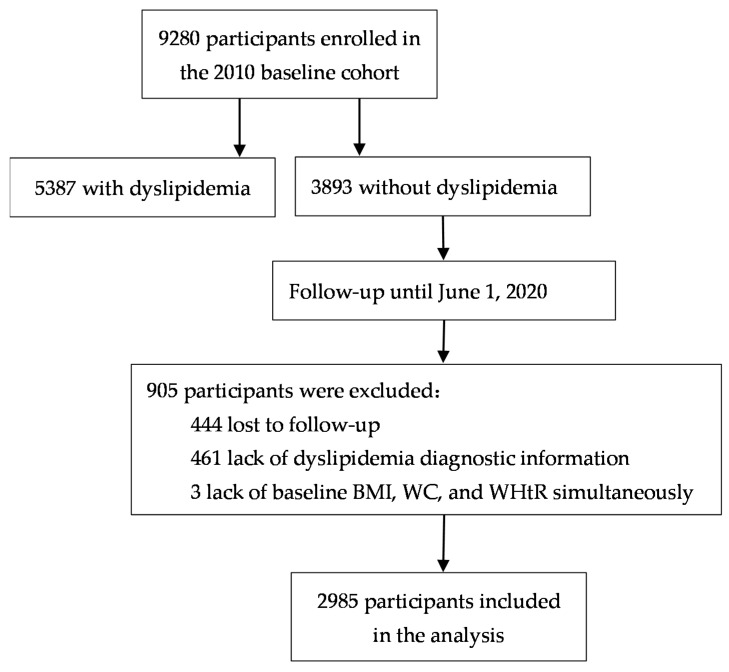
Flow chart for selection of study population.

The data used in this study were from the Guizhou Population Health Cohort Study (GPHCS), a prospective community-based cohort in Guizhou province, China [22]. Participants were recruited through a multi-stage cluster random sampling method. The baseline survey was carried out between November 2010 and December 2012, and a subsequent follow-up was conducted between December 2016 and June 2020. A total of 9280 adult residents from 48 townships of 12 districts in Guizhou province were recruited into this cohort. The inclusion criteria for this analysis included: (a) aged 18 years or above without any dyslipidemia at baseline; (b) at least one variate of BMI, WC, and WHtR at baseline without missing values; (c) completed the questionnaire and blood sample collection; (d) successfully followed-up. We further excluded 5387 individuals with dyslipidemia at baseline, 444 lost to follow-up, 461 with lack of dyslipidemia diagnostic information at follow-up, and 3 individuals missing baseline BMI, WC, and WHtR simultaneously. Finally, the remaining 2985 participants were eligible for the analysis (Figure 1). This study was approved by the Institutional Review Board of Guizhou Province Centre for Disease Control and Prevention (No. S2017-02), and written informed consent was obtained from all participants.

### 2.2. Data Collection

Baseline information, including demographic characteristics (age, gender, nationality, education level, residence, and marital status), lifestyle (smoking status, alcohol use, and physical activity), and chronic diseases (hypertension, dyslipidemia, diabetes mellitus, and cardiovascular diseases) was collected by trained investigators using a structured questionnaire via face-to-face interview. The questionnaire was designed by the Chinese Center for Disease Control and Prevention [23] and applied in China’s chronic disease surveillance (2010) [24].

Anthropometric measurements were collected by trained health professionals with standard procedures, which have been described in detail elsewhere [22]. Height was measured to the nearest 0.1 cm without shoes using a standard stadiometer (TZG, SHKODAK MEDICAL, Wuxi, China). Weight was measured in subjects wearing light clothing to the nearest 0.1 kg using a calibrated digital scale (TC-200K, G & G, Shanghai, China). WC was measured to the nearest 0.1 cm at the midpoint between the lowest rib margin and the level of the anterior superior iliac crest by a flexible anthropometric tape (Torch shaped waist measure, CN). BMI was calculated as weight in kg divided by height in m squared and was divided into four groups: underweight (<18.5 kg/m^2^), normal weight (18.5–23.9 kg/m^2^), overweight (24–27.9 kg/m^2^), and obesity (≥28 kg/m^2^) [25]. WHtR was calculated as WC in cm divided by height in cm. General obesity was defined as a BMI ≥ 28 kg/m^2^ [26]. Abdominal obesity was determined if meeting one of the following criteria: (a) WC ≥ 90 cm for men, and ≥85 cm for women [11]; (b) WHtR ≥ 0.5 [27]. Change in BMI or WHtR was calculated as differences between BMI or WHtR at follow-up and the corresponding values at baseline. WHtR change was divided into five groups (no gain or gain of <0.02, gain of ≥0.02 to gain of <0.06, gain of ≥0.06 to gain of <0.12, gain of ≥0.12 to gain of <0.20, and gain of ≥0.20) and was represented by <0.02, [0.02, 0.06), [0.06, 0.12), [0.12, 0.20), and ≥0.20, respectively. BMI change was also divided into five groups (loss of >2 kg/m^2^, loss of ≤2 kg/m^2^ to gain of <2 kg/m^2^, gain of ≥2 kg/m^2^ to gain of <6 kg/m^2^, gain of ≥6 kg/m^2^ to gain of <12 kg/m^2^, and gain of ≥12 kg/m^2^) and was represented by <−2, [−2, 2), [2, 6), [6, 12), and ≥12, respectively.

Three consecutive blood pressure measurements were taken and the mean value of three readings was used. For each participant, blood pressure measurement was performed on the right upper arm after 5 min of rest, with the participant in a seated position, using an electronic sphygmomanometer (HBP-1300, OMRON, Liaoning, China). Venous blood samples were obtained after at least 8 h overnight fasting. Plasma glucose was detected by the hexokinase method within 4 h. After centrifugation, serums separated from the remaining blood samples were stored at −20 °C and transferred to Guizhou Center for Disease Control and Prevention to detect the levels of total cholesterol (TC), high-density lipoprotein cholesterol (HDL-C), low-density lipoprotein cholesterol (LDL-C), and triglycerides (TG). All samples were analyzed with an autoanalyzer (Olympus 400 analyzer, Beckman Coulter, CA, USA).

All 9280 participants were followed up for the aforementioned information and vital status by a repeated investigation during 2016–2020, and 1117 (12.04%) were lost to follow-up. All deaths were confirmed through the Death Registration Information System and Basic Public Health Service System.

### 2.3. Definition

Current smoking referred to smoking tobacco products including manufactured or locally produced within the last 30 days [22]. Current alcohol drinking was defined as alcohol intake more than once a month during the past 12 months [24]. The physical activity information was collected by the International Physical Activity Questionnaire (IPAQ), which is publicly available online [28]. Regular physical exercise was considered as having physical activities at least three times a week (≥30 min each time) [29]. Hypertension was considered with systolic blood pressure (SBP) ≥ 140 mmHg and/or diastolic blood pressure (DBP) ≥ 90 mmHg, or self-reported hypertension or using antihypertensive medication [30]. Diabetes mellitus (DM) was defined if any of the following conditions were met [31]: fasting blood glucose ≥ 7.0 mmol/L, 2-h post-meal blood glucose ≥ 11.1 mmol/L, glycosylated hemoglobin ≥ 6.5%, self-reported diabetes diagnosed by doctors or receiving hyperglycemic treatment. CVD was considered with self-reported cardiovascular or cerebrovascular diseases by doctors or the primary cause of death was myocardial infarction, cerebral hemorrhage, cerebral infarction, or cerebral infarction. Dyslipidemia was diagnosed if participants had one or more of the following conditions [32]: TC ≥ 6.22 mmol/L, TG ≥ 2.26 mmol/L, HDL-C < 1.04 mmol/L, LDL-C ≥ 4.14 mmol/L, or self-reported dyslipidemia diagnosed by doctors or using lipid-lowering drugs.

### 2.4. Statistical Analysis

Continuous variables were expressed as means and standard deviations (SDs) and categorical variables as frequencies with proportions. They were compared using independent *t*-tests and *χ*^2^ tests, respectively. Person-years were calculated from the completion of the baseline investigation to the confirmation of mortality, incidence dyslipidemia, or 1 June 2020, whichever came first. Hazard ratio (HR), adjusted HR (aHR), and corresponding 95% confidential interval (CI) were estimated by Cox regression for associations between each anthropometric indicator and dyslipidemia. Potential covariates included age (less than 40 years, 40 to 59 years, 60 years or above) and sex (male or female) in Model 1, and additionally residence (rural or urban), nationality (Han Chinese or not), current alcohol drinking (yes or no) and regular physical exercise (yes or no) in Model 2. Baseline anthropometric indicators (categorical variables) were also adjusted for changes in corresponding variables. Test for linear trend was performed by extracting medians of categorical variables in the Cox regression model. Quantitative analyses were performed for per 5 kg/m^2^, 5 cm, 0.05, 5 kg/m^2^, 0.05 increment of BMI, WC, WHtR, changes in BMI and WHtR, respectively. We also qualitatively assessed the impact of changes in BMI and WHtR on the incidence of dyslipidemia. Subjects who were underweight at baseline and/or follow-up were excluded from the analysis of BMI change. We tested the interaction between all target variables and adjustment variables, and further conducted stratified analyses if significant interactions were observed. Finally, several sensitivity analyses were performed after excluding participants followed up less than three years, died with an unclear status of dyslipidemia, and having missing values of any covariant. All statistical procedures were performed using R version 4.0.3 (10 October 2020). All reported *p* values were 2-sided and <0.05 was considered significant.

## 3. Results

### 3.1. Baseline Characteristics of Participants

The number of total person-years (PYs) of follow-up was 20,926.7, and the mean duration of follow-up was 7.0 ± 1.1 years. Among 2985 eligible subjects, the average of age was 44.4 years old ranged from 18 to 89 years and more than half were women. During the follow-up, there were 2089 new-onset dyslipidemia events with an incidence of 99.8 per 1000 PYs. Subjects who developed dyslipidemia were more likely to be female, non-Han Chinese, non-smokers, non-drinkers, regular exercisers, patients without diabetes mellitus, living in urban regions, and having lower BMI and systolic BP (seen in Table 1).

### 3.2. Baseline WC, WHtR, and BMI and Risk of Incident Dyslipidemia

Table 2 shows the associations between different obesity-related anthropometric indices and the risk of incident dyslipidemia. Each 5-unit increment of baseline WC, WHtR, and BMI were associated with 4%, 7%, and 14% risk for incident dyslipidemia, respectively. Higher WHtR (≥0.5) or BMI (≥28.0 kg/m^2^) were significantly associated with a higher risk of dyslipidemia compared with WHtR of less than 0.5 or BMI of 18.5–23.9 kg/m^2^, respectively and a linear trend for HRs of BMI was detected (*p* for trend = 0.014). After the adjustment for age and sex, the association between WHtR, BMI, and the risk of incident dyslipidemia remained significant. Obesity subjects (BMI ≥ 28.0 kg/m^2^) still had a 45% higher risk of incident dyslipidemia than normal subjects (BMI 18.5–23.9 kg/m^2^) after the adjustment for age, sex, residence, nationality, current alcohol drinking, and regular physical exercise, while each 5.0 kg/m^2^ increment and the trend for aHRs of BMI were marginally associated with the incident risk.

### 3.3. Risk of Incident Dyslipidemia Due to Changes of WHtR and BMI

Table 3 shows the associations between changes of WHtR and BMI from baseline to follow-up and the risk of incident dyslipidemia. Compared with participants whose WHtR did not or slightly increased (WHtR change < 0.02), the risk of incident dyslipidemia over categories of WHtR increments (≥0.06 and <0.12, ≥0.12 and <0.20, ≥0.20) significantly increased, and a linear trend for HRs of WHtR change was observed (*p* for trend < 0.001). Per 0.05 increment of WHtR change was associated with 16% increased dyslipidemia risk. Compared with individuals who maintained a stable BMI (BMI change within −2.0 to 1.9 kg/m^2^), the risk of incident dyslipidemia significantly decreased among individuals with >2.0 kg/m^2^ loss of BMI, whereas it significantly increased among participants with BMI increments of 6–11.9 kg/m^2^ and ≥12 kg/m^2^ and there was a significant linear trend for HRs of BMI change (*p* for trend < 0.001). Each 5.0 kg/m^2^ increment of BMI change was associated with a 27% increased risk of dyslipidemia. The aforementioned HRs increased slightly after the adjustment of covariates. Additionally, subjects with BMI increments of 2–5.9 kg/m^2^ from baseline to follow-up showed a statistically increased risk of incident dyslipidemia (aHR = 1.21, 95%CI 1.02, 1.44).

**Figure 2:**
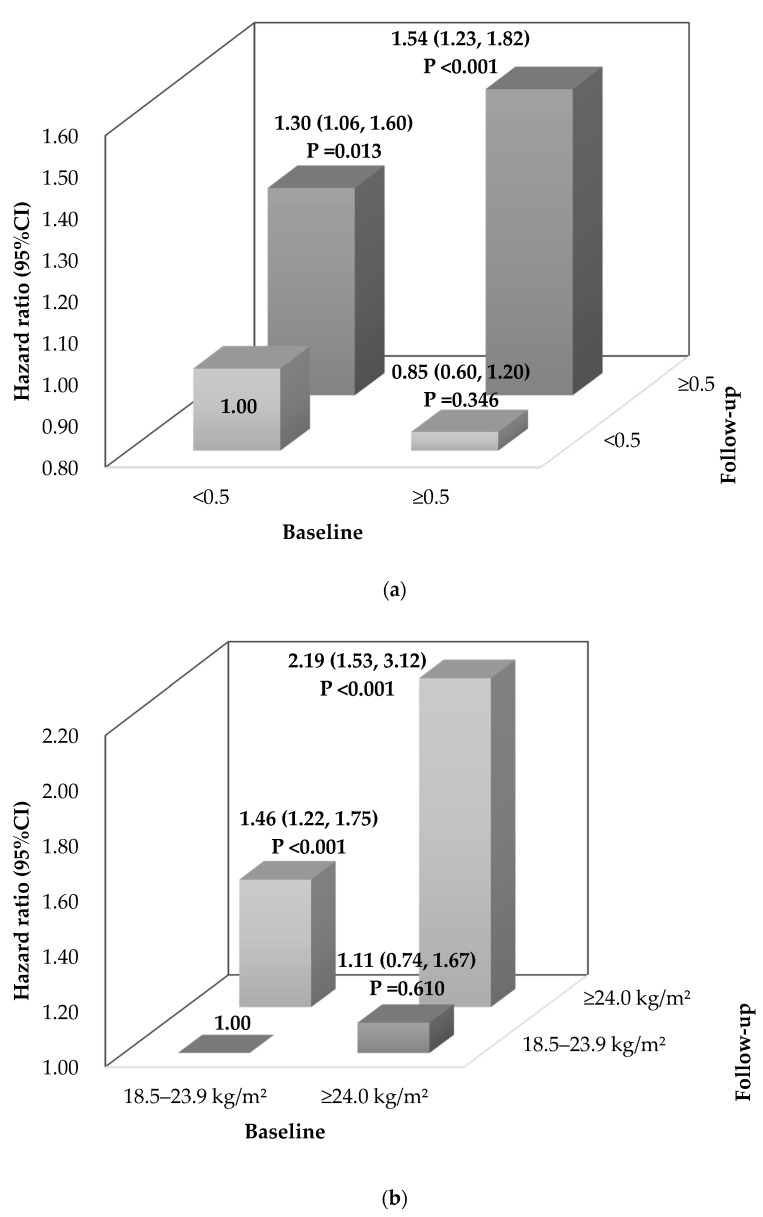
Adjusted hazard ratios (95% confidence intervals) of dyslipidemia associated with joint classification of WHtR and BMI categories from baseline to follow-up. (**a**) Joint classification of WHtR categories from baseline to follow-up; (**b**) Joint classification of BMI categories from baseline to follow-up. Note: Adjusted for age (categorical variable), sex, residence, nationality, current alcohol drinking, regular physical exercise, baseline WHtR categories (only for joint classification of WHtR categories), and baseline BMI categories (only for joint classification of BMI categories).

The attributable fraction [33] for incident dyslipidemia in the group of persistent abdominal obesity (WHtR ≥ 0.5) and the group with normal WHtR at baseline who developed abdominal obesity was 35.1% (18.7–45.1) and 23.1% (5.7–37.5), respectively (Figure 2). Compared with participants who maintained normal BMI, those who changed from normal to overweight/obesity (BMI ≥ 24 kg/m^2^) had an elevated risk of incident dyslipidemia (aHR = 1.46, 95%CI 1.22, 1.75), and those who maintained overweight/obesity at both time-points had the highest risk of dyslipidemia (aHR = 2.19, 95%CI 1.53, 3.12).

### 3.4. Stratification Analysis

Stratification analysis in Table 4 showed that age groups modified the associations of incident dyslipidemia with WC and WHtR (*p* for interaction = 0.015 and 0.030, respectively). Elders (≥60 years) showed a stronger association between WC and incident dyslipidemia, whereas significant association between WHtR and incident dyslipidemia was only found among subjects aged 40–59. For the same level of BMI gain, urban residents have a higher risk of incident dyslipidemia than rural residents (*p* for interaction = 0.045). For subjects developing abdominal obesity or persisting overweight/obesity, the risk of incident dyslipidemia was higher for male than that for female (Supplementary Figure S1a–d). Additionally, participants aged 40 years or above had higher aHRs of incident dyslipidemia for WHtR change and BMI change than those under 40 years (Supplementary Figure S1e–h). However, among those who developed overweight/obesity, the risk of incident dyslipidemia was stronger among participants ≥ 40 years than those <40 years (Supplementary Figure S1g,h).

### 3.5. Sensitivity Analysis

The findings were similar after the exclusion of subjects who were followed up less than three years or died with an unclear status of dyslipidemia (Supplementary Figures S2 and S3). Additionally, after the exclusion of subjects who have missing values of any covariant, the results were robust (Supplementary Figure S4).

## 4. Discussion

This prospective cohort study in Southwest China aimed to assess the effects of different anthropometric indices, their changes, and possible interactions between them and socioeconomic factors on the risk of incident dyslipidemia. Findings suggested that both WHtR and BMI were positively associated with the development of dyslipidemia, and both becoming obese and consistent obesity, whether general obesity or abdominal obesity, were notable risk factors for incident dyslipidemia among Chinese adults, especially among male and participants aged 40 years or above. Furthermore, dose-response relationships were found between BMI, BMI change, WHtR change, and the risk of incident dyslipidemia. Moreover, the associated risks were higher for those subjects who were middle-aged, elderly, male, and urban residents with poor control of obesity.

Previous studies have testified the association between dyslipidemia and various obesity indices among the Chinese population using cross-sectional designs [13,15,34,35]. In this prospective cohort study, the aHRs of incident dyslipidemia were increased with the increment of BMI and WHtR, which was similar to a recent study conducted in the US [17] and a study involving 44,048 Chinese [34]. Additionally, increased WC was associated with a higher risk of incident dyslipidemia, which was in line with previous studies [34,35]. However, this association was not statistically significant after adjusting for potential confounders. One possible explanation was that there was a relatively small sample size in this study because of the high prevalence of dyslipidemia. Another was that WC measurement was less reliable than weight and height [36]. Two studies conducted in northern China found that WC had a higher adjusted risk ratio/odds ratio of dyslipidemia than BMI [7,35], which was contrary to our results and another study conducted among Iranians [37]. Considering the variations in height and body size due to geographic and genetic differences, WC may be less useful than BMI to predict incident dyslipidemia among individuals with a relatively low height. Previous studies conducted in China [34], Spain [38], and other Asian countries [37,39] demonstrated that both general obesity and abdominal obesity showed significant associations with incident dyslipidemia. However, the best marker for anthropometric indices varied over different populations. Present study showed that marker of abdominal obesity measured by WHtR was more strongly related to dyslipidemia than WC among Chinese adults, which aligned with other studies conducted in China [34] and Spain [38]. This suggested that WHtR may be a better predictor than WC because it not only reflects body fat distribution and upper body adiposity but also accounts for differences in height [40], which improved the sensitivity in differentiating abdominal obesity subjects with the same WC but different height.

The traditional analytical methods above assessed the association of baseline anthropometric indices to the HRs for dyslipidemia, ensuring these indices were determined before the follow-up with the consideration that cause should be prior to effect [41]. However, Williams [42] suggested that the follow-up adiposity, rather than the baseline, determined the odds for hypercholesterolemia. Similar opinions have also been claimed in another study by Truesdale et al. [43]. Previous longitudinal studies mainly focused on changes in weight or WC in relation to incident dyslipidemia [2,44] or lipid profiles [20,36,42,45,46]. Here, we mainly focused on changes in WHtR and BMI because it was important to distinguish the risk of different subjects by taking height into account when they have comparable weight or WC changes. However, literature exploring the risk of dyslipidemia from the perspective of changes in BMI and WHtR was limited.

This study confirmed that both incident obesity and remaining obesity, whether general obesity or abdominal obesity, were notable risk factors for incident dyslipidemia, even after the adjustment of covariates, which was similar to previous findings [2]. In addition, dose-response relationships were found between the incidence of dyslipidemia and BMI change, as well as WHtR change, which indicated that losing BMI or WHtR may help reduce the dyslipidemia risk while gaining BMI or WHtR may increase such risk. We further targeted participants who were aged 40 years or above, male, or urban residents with poor control of obesity as a population at risk. Of note, Ishizaka et al. [36] reported that changes in BMI were positively associated with changes in LDL-C and TG, and negatively with those in HDL-C among Japanese. However, inverse association between BMI and cholesterol levels was also reported among some other populations [21]. This may be explained by a reduction in HDL cholesterol [47]. Changes in BMI may not be an exact surrogate marker of change in visceral fat area (VFA), because it was weakly correlated with the VFA change [48]. Therefore, changes in WHtR may be a predictor of incident dyslipidemia in this study.

Obesity-induced dyslipidemia has been identified as “metabolic-related dyslipidemia” [49], which is mainly driven by the effects of insulin resistance and proinflammatory adipokines [10]. Abundant evidence supports that weight loss is conducive to the improvement in lipid profile measures [46,50]. As an essential modifiable risk factor of CVD, systematic health education and early interventions should be recommended to help individuals with the above risk factors to lose weight and reduce CVD risk, especially among the Chinese population in the context of rapid economic development and shifting lifestyles.

To our knowledge, this study was first on the risk of incident dyslipidemia based on different anthropometric parameters and their changes among the community Chinese population in southwest China. The main strength of this study was to simultaneously explore the effects of several anthropometric indices and their changes on the risk of incident dyslipidemia with the 10-year follow-up. Additionally, those findings were robust after the sensitivity analysis. However, several limitations of this study should be noted. First, subjects were limited to adults in the Guizhou province of China, thus caution should be exercised in generalizing our findings to other populations. Second, the sample size was relatively small to explore the association between indices and incident dyslipidemia in smaller subgroups such as the elderly, retirees, and weight-losers. This also led to a conservative grouping strategy for WHtR change in order to avoid violating the proportional hazard assumption. Thus, the protective effect of WHtR loss for incident dyslipidemia could not be evaluated in this study. Third, the impact of family dyslipidemia was not well differentiated in this study. In addition, anthropometric measurements in this study were not able to reflect the distribution and proportion of body fat in reality. Our findings called for better-designed studies with large sample size in different populations using the measures of the real body fat composition in the future. Additionally, hereditary dyslipidemia requires more attention in the future research.

## 5. Conclusions

In conclusion, this long-term prospective study demonstrated that BMI and WHtR had positive effects on the risk of incident dyslipidemia in Chinese adults. General obesity, abdominal obesity, excessive gain in BMI or WHtR were considerable risk factors in developing dyslipidemia. Therefore, to control and reduce BMI or WHtR to the normal range through lifestyle modification are urgently needed for population at risk, especially for those who were aged 40 years or above, male, and urban residents with poor control of obesity.

## Figures and Tables

**Table 1:** Baseline characteristics of subjects from this cohort by incident dyslipidemia (%).

Characteristics	Total (*n* = 2985)	Dyslipidemia (*n* = 2089)	Non-Dyslipidemia (*n* = 896)	*p* Value
Female	1547 (51.8)	1126 (53.9)	421 (47.0)	0.001
Age, years	44.43 ± 15.44	44.27 ± 15.44	44.82 ± 15.43	0.365
Non-Han Chinese	1634 (54.7)	1172 (56.1)	462 (51.6)	0.025
Rural residents	1921 (64.4)	1294 (61.9)	627 (70.0)	<0.001
Married	542 (18.2)	378 (18.1)	164 (18.3)	0.933
Nine education years or longer	1933 (64.8)	1362 (65.2)	571 (63.7)	0.466
Current smoking	801 (26.8)	524 (25.1)	277 (30.9)	0.001
Current alcohol drinking *	884 (29.6)	565 (27.1)	319 (35.6)	<0.001
Regular physical exercise	1194 (40.0)	863 (41.3)	331 (36.9)	0.028
Hypertension *	636 (21.3)	433 (20.7)	203 (22.7)	0.258
Diabetes mellitus *	161 (5.4)	100 (4.8)	61 (6.9)	0.030
Cardiovascular disease *	21 (0.7)	14 (0.7)	7 (0.8)	0.923
Waist circumference, cm *	74.88 ± 8.63	74.68 ± 8.59	75.32 ± 8.69	0.065
≥85/90	280 (9.5)	187 (9.1)	93 (10.6)	0.237
BMI, kg/m^2^	22.21 ± 3.00	22.11 ± 2.98	22.42 ± 3.03	0.011
<18.5	208 (7.0)	156 (7.5)	52 (5.8)	0.115
18.5-	2099 (70.3)	1472 (70.5)	627 (70.0)	
24.0-	567 (19.0)	392 (18.8)	175 (19.5)	
≥28.0	111 (3.7)	69 (3.3)	42 (4.7)	
WHtR *	0.48 ± 0.06	0.48 ± 0.06	0.48 ± 0.06	0.104
≥0.5	918 (31.2)	621 (30.1)	297 (33.7)	0.062
SBP, mmHg *	120.91 ± 19.63	120.08 ± 19.30	122.85 ± 20.25	<0.001
DBP, mmHg *	76.38 ± 11.47	76.22 ± 11.40	76.74 ± 11.63	0.258
FPG, mmol/L *	5.19 ± 1.05	5.18 ± 1.07	5.21 ± 1.02	0.573
TC, mg/dL *	5.79 ± 0.38	5.85 ± 0.37	5.75 ± 0.39	0.054
TG, mg/dL *	4.38 ± 3.68	4.49 ± 4.29	4.31 ± 3.20	0.699
HDL-C, mg/dL *	4.70 ± 0.77	4.71 ± 0.78	4.67 ± 0.77	0.127
LDL-C, mg/dL *	1.16 ± 0.44	1.15 ± 0.44	1.17 ± 0.45	0.317

Abbreviations: BMI, body mass index; WHtR, waist-to-height ratio; FPG, fasting plasma glucose; SBP, systolic blood pressure; DBP, diastolic blood pressure; TC, total cholesterol; TG, triglyceride; HDL-C, high-density lipoprotein cholesterol; LDL-C, low-density lipoprotein cholesterol. * Missing value exists.

**Table 2:** Associations between baseline WC, WHtR, BMI, and the risk of incident dyslipidemia.

Variables	Cases, *n*	ID/1000 PYs	HR (95%CI)	*p* Value	aHR * (95%CI)	*p* Value	aHR # (95%CI)	*p* Value
WC (per 5 cm increment)	881	42.66	1.04 (1.01, 1.08)	0.019	1.03 (0.99, 1.07)	0.112	1.02 (0.98, 1.06)	0.351
<85/90	788	42.15	1.00	-	1.00	-	1.00	-
≥85/90	93	47.53	1.17 (0.94, 1.45)	0.156	1.16 (0.94, 1.44)	0.170	1.12 (0.90, 1.39)	0.323
WHtR (per 0.05 increment)	881	42.66	1.07 (1.01, 1.13)	0.023	1.06 (1.00, 1.13)	0.043	1.04 (0.98, 1.11)	0.157
<0.5	584	41.03	1.00	-	1.00	-	1.00	-
≥0.5	297	46.27	1.16 (1.01, 1.33)	0.037	1.16 (1.01, 1.34)	0.039	1.13 (0.98, 1.31)	0.086
BMI (per 5.0 kg/m^2^ increment)	896	42.82	1.14 (1.03, 1.25)	0.009	1.12 (1.01, 1.23)	0.024	1.10 (1.00, 1.22)	0.053
<18.5	52	35.77	0.78 (0.59, 1.04)	0.089	0.80 (0.60, 1.06)	0.116	0.84 (0.63, 1.12)	0.246
18.5-	627	42.58	1.00	-	1.00	-	1.00	-
24.0-	175	43.85	1.00 (0.85, 1.19)	0.955	1.01 (0.85, 1.19)	0.947	1.00 (0.84, 1.19)	0.999
≥28.0	42	55.42	1.53 (1.12, 2.10)	0.007	1.49 (1.09, 2.04)	0.012	1.45 (1.05, 1.99)	0.023
*p* for trend				0.014		0.021		0.056

Note: * Adjusted for age (categorical variable), sex. # Adjusted for age (categorical variable), sex, residence, nationality, current alcohol drinking, and regular physical exercise. Abbreviations: ID incident density; PY, person years; HR, hazard ratio; 95%CI, 95% confidence interval; WC, waist circumference; BMI, body mass index; WHtR, waist-to-height ratio.

**Table 3:** Associations between changes of WHtR and BMI from baseline to follow-up and the risk of incident dyslipidemia.

Variables	Cases, *n*	ID/1000 PYs	HR (95%CI)	*p* Value	aHR * (95%CI)	*p* Value	aHR # (95%CI)	*p* Value
WHtR change (per 0.05 increase)	631	48.47	1.16 (1.10, 1.23)	<0.001	1.16 (1.10, 1.23)	<0.001	1.25 (1.18, 1.33)	<0.001
<0.02	211	45.31	1.00	-	1.00	-	1.00	-
[0.02, 0.06)	155	44.36	1.02 (0.83, 1.26)	0.817	1.00 (0.81, 1.23)	0.998	1.13 (0.91, 1.41)	0.267
[0.06, 0.12)	179	52.58	1.41 (1.16, 1.73)	0.001	1.39 (1.14, 1.70)	0.001	1.60 (1.29, 1.99)	<0.001
[0.12, 0.20)	70	57.07	1.63 (1.24, 2.14)	<0.001	1.59 (1.21, 2.08)	0.001	2.00 (1.49, 2.69)	<0.001
≥0.20	16	67.39	1.96 (1.18, 3.26)	0.009	2.07 (1.24, 3.45)	0.005	2.77 (1.63, 4.71)	<0.001
*p* for trend				<0.001		<0.001		<0.001
BMI change (per 5.0 kg/m^2^ increase)	783	45.61	1.27 (1.15, 1.39)	<0.001	1.30 (1.18, 1.42)	<0.001	1.43 (1.31, 1.57)	<0.001
<−2	105	37.71	0.78 (0.63, 0.97)	0.024	0.77 (0.62, 0.96)	0.019	0.73 (0.58, 0.91)	0.007
[−2, 2)	367	42.09	1.00	-	1.00	-	1.00	-
[2, 6)	225	50.79	1.09 (0.92, 1.29)	0.300	1.10 (0.93, 1.30)	0.258	1.21 (1.02, 1.44)	0.026
[6, 12)	77	67.75	1.43 (1.12, 1.83)	0.005	1.50 (1.17, 1.93)	0.001	1.86 (1.44, 2.41)	<0.001
*p* for trend				<0.001		<0.001		<0.001

Note: * Adjusted for age (categorical variable), sex. # Adjusted for age (categorical variable), sex, residence, nationality, current alcohol drinking, regular physical exercise, baseline BMI (categorical variable) for variables related to BMI change, and baseline WHtR (categorical variable) for variables related to WHtR change. Abbreviations: ID, incident density; PY, person years; HR, hazard ratio; 95%CI, 95% confidence interval; BMI, body mass index; WHtR, waist-to-height ratio.

**Table 4:** Stratification analysis of different anthropometric risk factors for incident dyslipidemia by age groups and residence.

Stratification Variable	Effect Variable	aHR (95%CI)	*p* Value
Age, years	WC		
<40	<85/90	1.00	-
	≥85/90	0.71 (0.46, 1.09)	0.118
40-	<85/90	1.00	-
	≥85/90	1.19 (0.87, 1.63)	0.268
≥60	<85/90	1.00	-
	≥85/90	1.92 (1.19, 3.07)	0.007
*p* for interaction			0.015
Age, years	WHtR		
<40	<0.5	1.00	-
	≥0.5	0.92 (0.72, 1.17)	0.512
40-	<0.5	1.00	-
	≥0.5	1.31 (1.06, 1.62)	0.014
≥60	<0.5	1.00	-
	≥0.5	1.12 (0.80, 1.56)	0.506
*p* for interaction			0.030
Rural residents	BMI change		
No	<−2	0.67 (0.43, 1.07)	0.093
	[−2, 2)	1.00	-
	[2, 6)	1.69 (1.22, 2.36)	0.002
	[6, 12)	3.26 (1.99, 5.33)	<0.001
	≥12	2.53 (0.77, 8.26)	0.126
Yes	<−2	0.71 (0.54, 0.93)	0.014
	[−2, 2)	1.00	-
	[2, 6)	1.07 (0.88, 1.31)	0.501
	[6, 12)	1.53 (1.13, 2.07)	0.006
	≥12	2.38 (0.99, 5.68)	0.052
*p* for interaction			0.045

Note: Adjusted for age (categorical variable), sex, residence, nationality, current alcohol drinking, regular physical exercise, and baseline BMI (categorical variable) for BMI change. Abbreviations: aHR, adjusted hazard ratio; 95%CI, 95% confidence interval; WC, waist circumference; BMI, body mass index; WHtR, waist-to-height ratio.

## Data Availability

The datasets generated for this study are available on request to the corresponding author.

## Supplementary Materials

The following are available online at https://www.mdpi.com/article/10.3390/ijerph19010341/s1, Figure S1: Adjusted hazard ratios (95% confidence intervals) of dyslipidemia associated with joint classification of WHtR and BMI categories from baseline to follow-up by age groups and sex; Figure S2: Sensitivity analysis after excluding participants who were followed-up less than three years (2965 remained); Figure S3: Sensitivity analysis after excluding participants who died with unclear status of dyslipidemia (2945 remained); Figure S4: Sensitivity analysis after excluding participants who has missing values of any covariant (1924 remained).

## References

[1] Kopin L., Lowenstein C. (2017). Dyslipidemia. Ann. Intern. Med..

[2] Zhou J., Ren Y., Wang C., Li L., Zhang L., Wang B., Zhao Y., Han C., Zhang H., Yang X. (2018). Association of change in waist circumference and dyslipidaemia risk: The rural Chinese cohort study. Diabetes Metab. Res. Rev..

[3] Cleeman J., Grundy S., Becker D., Clark L., Cooper R., Denke M., Howard W., Hunninghake D., Illingworth D. (2001). Executive Summary of The Third Report of The National Cholesterol Education Program (NCEP) Expert Panel on Detection, Evaluation, and Treatment of High Blood Cholesterol in Adults (Adult Treatment Panel III). JAMA.

[4] Roth G.A., Johnson C., Abajobir A., Abd-Allah F., Abera S.F., Abyu G., Ahmed M., Aksut B., Alam T., Alam K. (2017). Global, Regional, and National Burden of Cardiovascular Diseases for 10 Causes, 1990 to 2015. J. Am. Coll. Cardiol..

[5] Ma L.Y., Chen W.W., Gao R.L., Liu L.S., Zhu M.L., Wang Y.J., Wu Z.S., Li H.J., Gu D.F., Yang Y.J. (2020). China cardiovascular diseases report 2018: An updated summary. J. Geriatr. Cardiol..

[6] Opoku S., Gan Y., Yobo E.A., Tenkorang-Twum D., Yue W., Wang Z., Lu Z. (2021). Awareness, treatment, control, and determinants of dyslipidemia among adults in China. Sci. Rep..

[7] Xi Y., Niu L., Cao N., Bao H., Xu X., Zhu H., Yan T., Zhang N., Qiao L., Han K. (2020). Prevalence of dyslipidemia and associated risk factors among adults aged ≥35 years in northern China: A cross-sectional study. BMC Public Health.

[8] Lee J.S., Chang P.Y., Zhang Y., Kizer J.R., Best L.G., Howard B.V. (2017). Triglyceride and HDL-C Dyslipidemia and Risks of Coronary Heart Disease and Ischemic Stroke by Glycemic Dysregulation Status: The Strong Heart Study. Diabetes Care.

[9] Pikula A., Beiser A.S., Wang J., Himali J.J., Kelly-Hayes M., Kase C.S., Yang Q., Seshadri S., Wolf P.A. (2015). Lipid and lipoprotein measurements and the risk of ischemic vascular events: Framingham Study. Neurology.

[10] Vekic J., Zeljkovic A., Stefanovic A., Jelic-Ivanovic Z., Spasojevic-Kalimanovska V. (2019). Obesity and dyslipidemia. Metabolism.

[11] Wang H., Liu A., Zhao T., Gong X., Pang T., Zhou Y., Xiao Y., Yan Y., Fan C., Teng W. (2017). Comparison of anthropometric indices for predicting the risk of metabolic syndrome and its components in Chinese adults: A prospective, longitudinal study. BMJ Open.

[12] Zhou Z., Li K., Li X., Luan R., Zhou R. (2021). Independent and joint associations of body mass index, waist circumference, waist-height ratio and their changes with risks of hyperuricemia in middle-aged and older Chinese individuals: A population-based nationwide cohort study. Nutr. Metab..

[13] Rao W., Su Y., Yang G., Ma Y., Liu R., Zhang S., Wang S., Fu Y., Kou C., Yu Y. (2016). Cross-Sectional Associations between Body Mass Index and Hyperlipidemia among Adults in Northeastern China. Int. J. Environ. Res. Public Health.

[14] Lo K., Huang Y.Q., Shen G., Huang J.Y., Liu L., Yu Y.L., Chen C.L., Feng Y.Q. (2021). Effects of waist to height ratio, waist circumference, body mass index on the risk of chronic diseases, all-cause, cardiovascular and cancer mortality. Postgrad. Med. J..

[15] Du S.M., Ma G.S., Li Y.P., Fang H.Y., Hu X.Q., Yang X.G., Hu Y.H. (2010). Relationship of body mass index, waist circumference and cardiovascular risk factors in Chinese adult. Biomed. Environ. Sci..

[16] Obirikorang C., Obirikorang Y., Acheampong E., Anto E.O., Toboh E., Asamoah E.A., Amakwaa B., Batu E.N., Brenya P. (2018). Association of Wrist Circumference and Waist-to-Height Ratio with Cardiometabolic Risk Factors among Type II Diabetics in a Ghanaian Population. J. Diabetes Res..

[17] Rangel-Baltazar E., Cuevas-Nasu L., Shamah-Levy T., Rodríguez-Ramírez S., Méndez-Gómez-Humarán I., Rivera J.A. (2019). Association between High Waist-to-Height Ratio and Cardiovascular Risk among Adults Sampled by the 2016 Half-Way National Health and Nutrition Survey in Mexico (ENSANUT MC 2016). Nutrients.

[18] Alzeidan R., Fayed A., Rabiee F., Hersi A., Elmorshedy H. (2020). Diagnostic performance of waist-to-height ratio in identifying cardiovascular risk factors and metabolic syndrome among adult Saudis. A cross-sectional study. Saudi Med. J..

[19] Jia G.C., Shu X.O., Liu Y., Li H.L., Cai H., Gao J., Gao Y.T., Wen W.Q., Xiang Y.B., Zheng W. (2019). Association of Adult Weight Gain with Major Health Outcomes Among Middle-aged Chinese Persons with Low Body Weight in Early Adulthood. JAMA Netw. Open.

[20] Balkau B., Picard P., Vol S., Fezeu L., Eschwège E. (2007). Consequences of change in waist circumference on cardiometabolic risk factors over 9 years: Data from an Epidemiological Study on the Insulin Resistance Syndrome (DESIR). Diabetes Care..

[21] Van Hemelrijck M., Ulmer H., Nagel G., Peter R.S., Fritz J., Myte R., van Guelpen B., Föger B., Concin H., Häggström C. (2018). Longitudinal study of body mass index, dyslipidemia, hyperglycemia, and hypertension in 60,000 men and women in Sweden and Austria. PLoS ONE.

[22] Yu Y., Chen Y., Wang Y., Yu L., Liu T., Fu C. (2021). Is the Efficiency Score an Indicator for Incident Hypertension in the Community Population of Western China?. Int. J. Environ. Res. Public Health.

[23] Zhao W.H., Ning G. (2012). Contents and methods of chronic disease surveillance project in China in 2010. Chin. J. Prev. Med..

[24] Xu Y., Wang L., He J., Bi Y., Li M., Wang T., Wang L., Jiang Y., Dai M., Lu J. (2013). Prevalence and Control of Diabetes in Chinese Adults. JAMA.

[25] Wang J., Taylor A.W., Zhang T., Appleton S., Shi Z. (2018). Association between Body Mass Index and All-Cause Mortality among Oldest Old Chinese. J. Nutr. Health Aging.

[26] Ashwell M., Lejeune S., McPherson K. (1996). Ratio of waist circumference to height may be better indicator of need for weight management. BMJ.

[27] Browning L.M., Hsieh S.D., Ashwell M. (2010). A systematic review of waist-to-height ratio as a screening tool for the prediction of cardiovascular disease and diabetes: 0·5 could be a suitable global boundary value. Nutr. Res. Rev..

[28] (2015). The IPAQ Group Guidelines for Data Processing and Analysis of the International Physical Activity Questionnaire. http://www.ipaq.ki.se.

[29] Prazeres L., Brito R., Silva É. (2018). Regular physical exercise, sedentarism and characteristics of dismenorrhea and premenstrual syndrome. Fisioter. Mov..

[30] Liu L.S., Revision J.C.f.G. (2019). 2018 Chinese Guidelines for Prevention and Treatment of Hypertension-A report of the Revision Committee of Chinese Guidelines for Prevention and Treatment of Hypertension. J. Geriatr. Cardiol..

[31] Jia W., Weng J., Zhu D., Ji L., Lu J., Zhou Z., Zou D., Guo L., Ji Q., Chen L. (2019). Standards of medical care for type 2 diabetes in China 2019. Diabetes Metab. Res. Rev..

[32] Zhu J.R., Gao R.L., Zhao S.P., Lu G.P., Zhao D., Li J.J. (2018). 2016 Chinese guidelines for the management of dyslipidemia in adults. J. Geriatr. Cardiol..

[33] Lam T.H. (2012). Absolute risk of tobacco deaths: One in two smokers will be killed by smoking: Comment on “Smoking and all-cause mortality in older people”. Arch. Intern. Med..

[34] Liu J., Tse L.A., Liu Z., Rangarajan S., Hu B., Yin L., Leong D.P., Li W. (2019). Predictive Values of Anthropometric Measurements for Cardiometabolic Risk Factors and Cardiovascular Diseases Among 44,048 Chinese. J. Am. Heart Assoc..

[35] Feng R.N., Zhao C., Wang C., Niu Y.C., Li K., Guo F.C., Li S.T., Sun C.H., Li Y. (2012). BMI is strongly associated with hypertension, and waist circumference is strongly associated with type 2 diabetes and dyslipidemia, in northern Chinese adults. J. Epidemiol..

[36] Ishizaka N., Ishizaka Y., Toda E., Koike K., Nagai R., Yamakado M. (2009). Impact of changes in waist circumference and BMI over one-year period on serum lipid data in Japanese individuals. J. Atheroscler. Thromb..

[37] Ebrahimi H., Emamian M.H., Hashemi H., Fotouhi A. (2016). Dyslipidemia and its risk factors among urban middle-aged Iranians: A population-based study. Diabetes Metab. Syndr..

[38] Sangrós F.J., Torrecilla J., Giráldez-García C., Carrillo L., Mancera J., Mur T., Franch J., Díez J., Goday A., Serrano R. (2018). Association of General and Abdominal Obesity With Hypertension, Dyslipidemia and Prediabetes in the PREDAPS Study. Rev. Esp. Cardiol..

[39] Shirasawa T., Ochiai H., Yoshimoto T., Nagahama S., Kobayashi M., Ohtsu I., Sunaga Y., Kokaze A. (2019). Associations between normal weight central obesity and cardiovascular disease risk factors in Japanese middle-aged adults: A cross-sectional study. J. Health Popul. Nutr..

[40] Zeng Q., He Y., Dong S., Zhao X., Chen Z., Song Z., Chang G., Yang F., Wang Y. (2014). Optimal cut-off values of BMI, waist circumference and waist: Height ratio for defining obesity in Chinese adults. Br. J. Nutr..

[41] Petersen L., Schnohr P., Sørensen T.I. (2004). Longitudinal study of the long-term relation between physical activity and obesity in adults. Int. J. Obes. Relat. Metab. Disord..

[42] Williams P.T. (2008). Changes in body weight and waist circumference affect incident hypercholesterolemia during 7 years of follow-up. Obesity.

[43] Truesdale K.P., Stevens J., Lewis C.E., Schreiner P.J., Loria C.M., Cai J. (2006). Changes in risk factors for cardiovascular disease by baseline weight status in young adults who maintain or gain weight over 15 years: The CARDIA study. Int. J. Obes..

[44] Sogabe N., Sawada S.S., Lee I.M., Kawakami R., Ishikawa-Takata K., Nakata Y., Mitomi M., Noguchi J., Tsukamoto K., Miyachi M. (2016). Weight change after 20 years of age and the incidence of dyslipidemia: A cohort study of Japanese male workers. J. Public Health.

[45] Norman J.E., Bild D., Lewis C.E., Liu K., West D.S. (2003). The impact of weight change on cardiovascular disease risk factors in young black and white adults: The CARDIA study. Int. J. Obes..

[46] Hasegawa Y., Nakagami T., Oya J., Takahashi K., Isago C., Kurita M., Tanaka Y., Ito A., Kasahara T., Uchigata Y. (2019). Body Weight Reduction of 5% Improved Blood Pressure and Lipid Profiles in Obese Men and Blood Glucose in Obese Women: A Four-Year Follow-up Observational Study. Metab. Syndr. Relat. Disord..

[47] Rashid S., Genest J. (2007). Effect of obesity on high-density lipoprotein metabolism. Obesity.

[48] Matsushita Y., Nakagawa T., Yamamoto S., Takahashi Y., Yokoyama T., Mizoue T., Noda M. (2012). Effect of longitudinal changes in visceral fat area and other anthropometric indices to the changes in metabolic risk factors in Japanese men: The Hitachi Health Study. Diabetes Care.

[49] Su X., Cheng Y., Zhang G., Wang B. (2021). Novel insights into the pathological mechanisms of metabolic related dyslipidemia. Mol. Biol. Rep..

[50] Rosenkilde M., Rygaard L., Nordby P., Nielsen L.B., Stallknecht B. (2018). Exercise and weight loss effects on cardiovascular risk factors in overweight men. J. Appl. Physiol..

