# Supplementary material for: Effects of Body Mass Index, Waist Circumference, Waist-to-Height Ratio and Their Changes on Risks of Dyslipidemia among Chinese Adults: The Guizhou Population Health Cohort Study
Source: Int J Environ Res Public Health. 2021 Dec 29;19(1):341. doi: 10.3390/ijerph19010341 (PMC8750900; doi:10.3390/ijerph19010341)
Supplement: Supplementary file 1 [file ijerph-19-00341-s001.zip › ijerph-1506675-supplementary.pdf]

International Journal of
Environmental Research and Public Health

MDPI

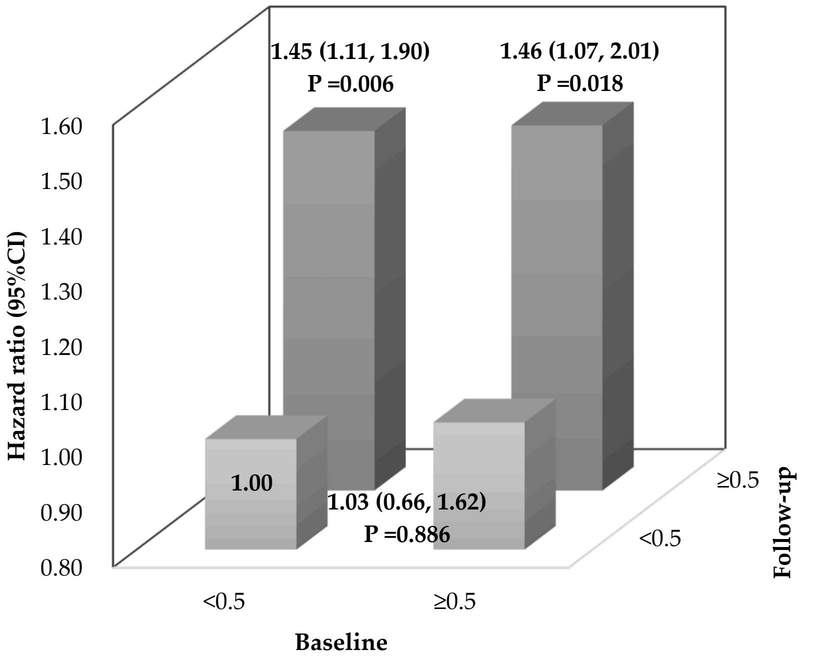

(a)

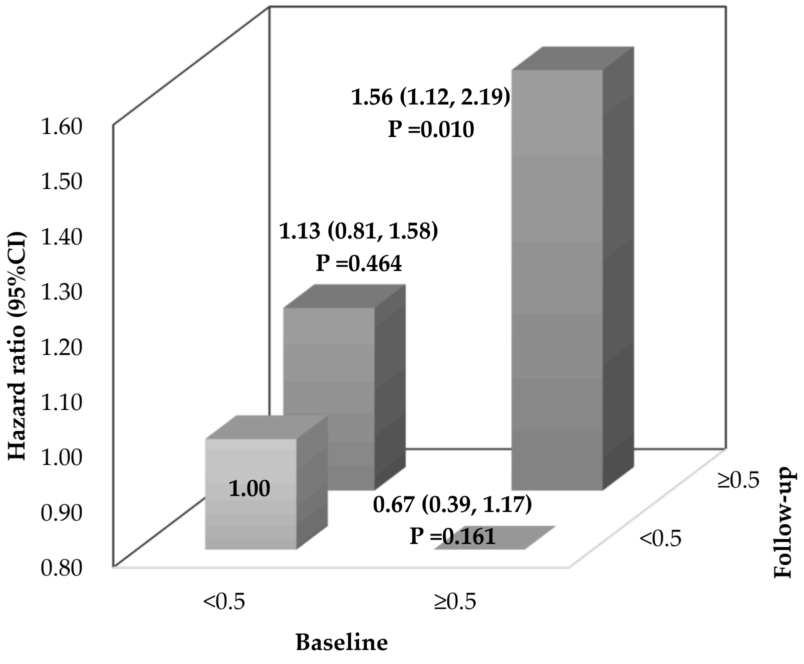

(b)

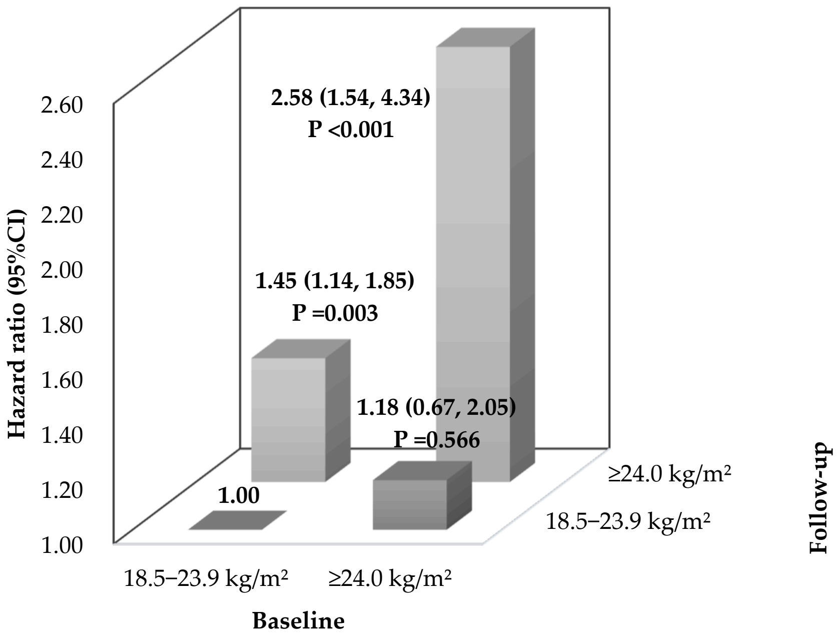

(c)

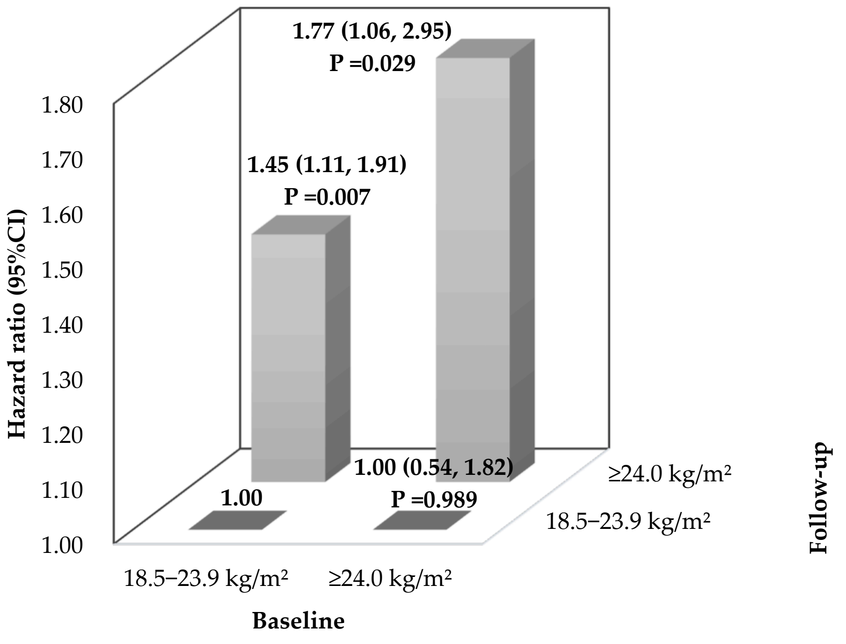

(d)

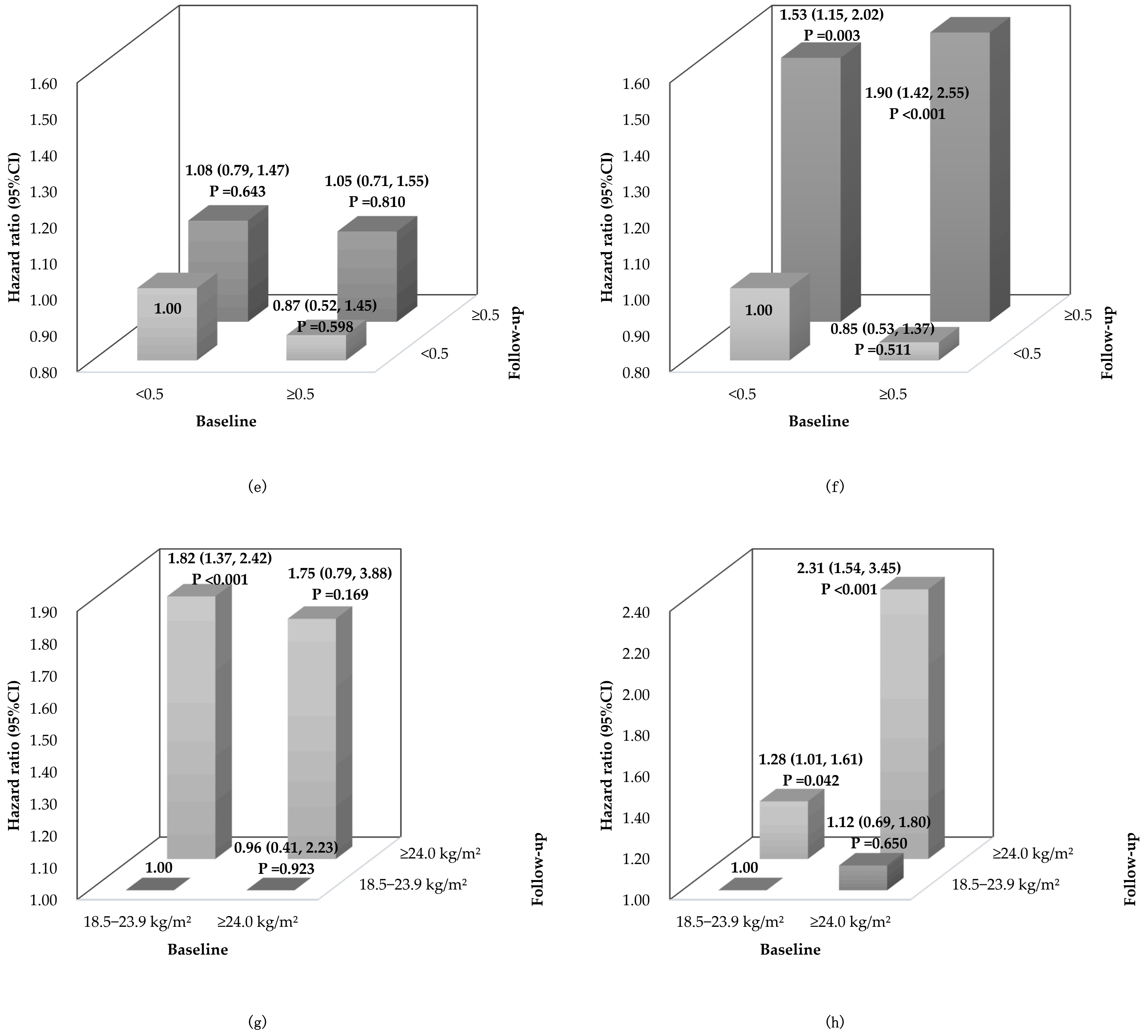

Figure S1. Adjusted hazard ratios (95% confidence intervals) of dyslipidemia associated with joint classification of WHtR and BMI categories from baseline to follow-up by age groups and sex. (a) Joint classification of WHtR categories from baseline to follow-up among male; (b) Joint classification of WHtR categories from baseline to follow-up among female; (c) Joint classification of BMI categories from baseline to follow-up among male; (d) Joint classification of BMI categories from baseline to follow-up among female; (e) Joint classification of WHtR categories from baseline to follow-up among participants <40 years; (f) Joint classification of WHtR categories from baseline to follow-up among participants ≥40 years; (g) Joint classification of BMI categories from baseline to follow-up among participants <40 years; (h) Joint classification of BMI categories from baseline to follow-up among participants ≥40 years. Note: Adjusted for age (categorical variable), sex, residence, nationality, current alcohol drinking, regular physical exercise, baseline WHtR categories (only for joint classification of WHtR categories), and baseline BMI categories (only for joint classification of BMI categories).

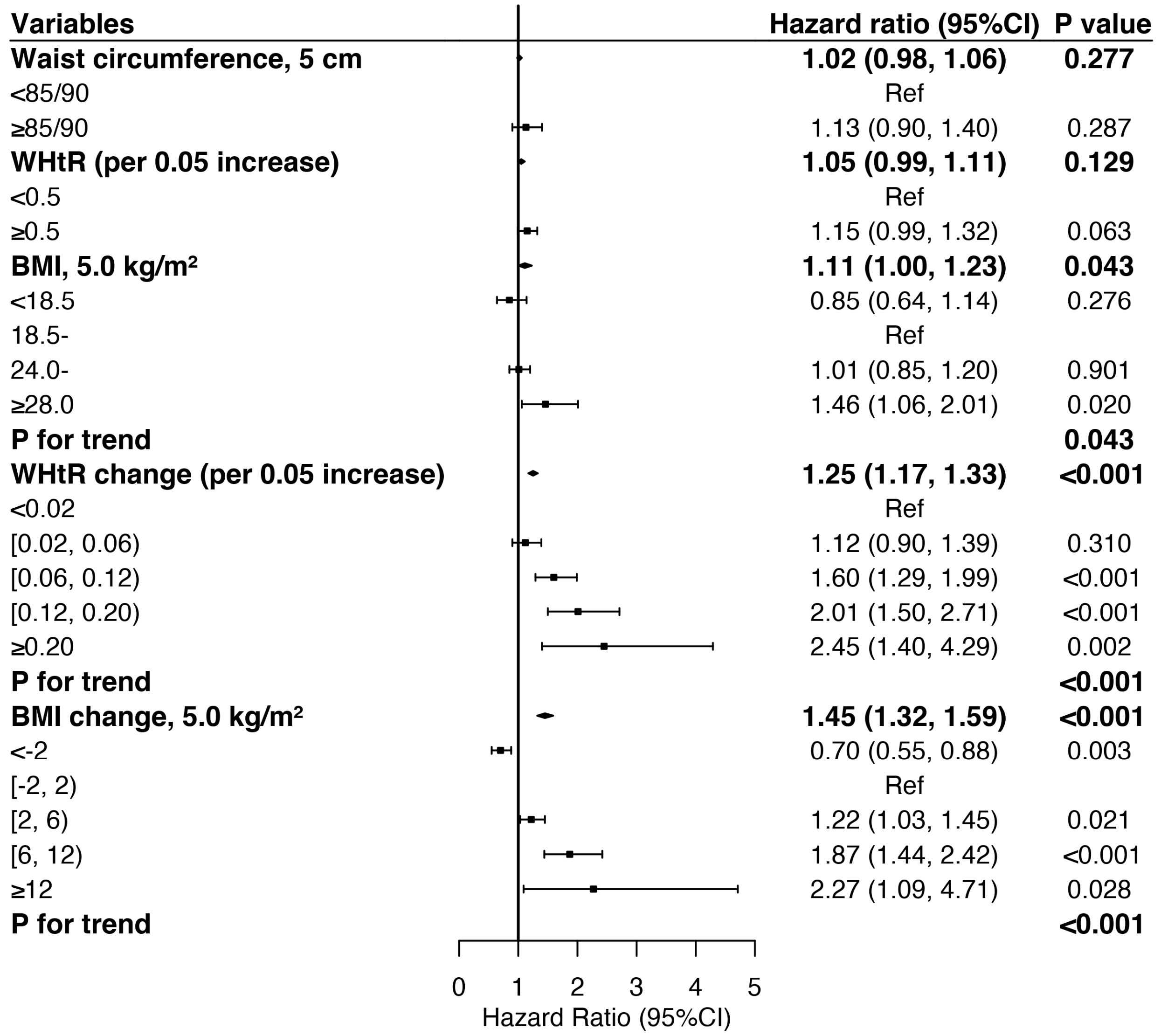

Figure. S2 Sensitivity analysis after excluding participants who were followed-up less than three years (2965 remained). HR hazard ratio; 95%CI 95% confidence interval; BMI body mass index; WHtR waist-to-height ratio. **Note:** Adjusted for age (categorical variable), sex, residence, nationality, current alcohol consumption, regular physical exercise, baseline BMI (categorical variable) for variables related to BMI change, and baseline WHtR (categorical variable) for variables related to WHtR change.

Variables	Hazard ratio (95%CI)	P value
**Waist circumference, 5 cm**	**1.02 (0.98, 1.06)**	**0.363**
<85/90	Ref	
≥85/90	1.12 (0.90, 1.39)	0.329
**WHtR (per 0.05 increase)**	**1.04 (0.98, 1.11)**	**0.159**
<0.5	Ref	
≥0.5	1.13 (0.98, 1.31)	0.088
**BMI, 5.0 kg/m²**	**1.10 (0.99, 1.22)**	**0.063**
<18.5	0.85 (0.64, 1.13)	0.266
18.5-	Ref	
24.0-	1.00 (0.84, 1.18)	0.969
≥28.0	1.45 (1.05, 1.99)	0.023
**P for trend**		**0.058**
**WHtR change (per 0.05 increase)**	**1.25 (1.18, 1.33)**	**<0.001**
<0.02	Ref	
[0.02, 0.06)	1.13 (0.91, 1.41)	0.267
[0.06, 0.12)	1.60 (1.29, 1.99)	<0.001
[0.12, 0.20)	2.00 (1.49, 2.69)	<0.001
≥0.20	2.77 (1.63, 4.71)	<0.001
**P for trend**		**<0.001**
**BMI change, 5.0 kg/m²**	**1.45 (1.32, 1.59)**	**<0.001**
<-2	0.69 (0.55, 0.87)	0.002
[-2, 2)	Ref	
[2, 6)	1.21 (1.02, 1.44)	0.028
[6, 12)	1.87 (1.44, 2.42)	<0.001
≥12	2.53 (1.27, 5.07)	0.008
**P for trend**		**<0.001**

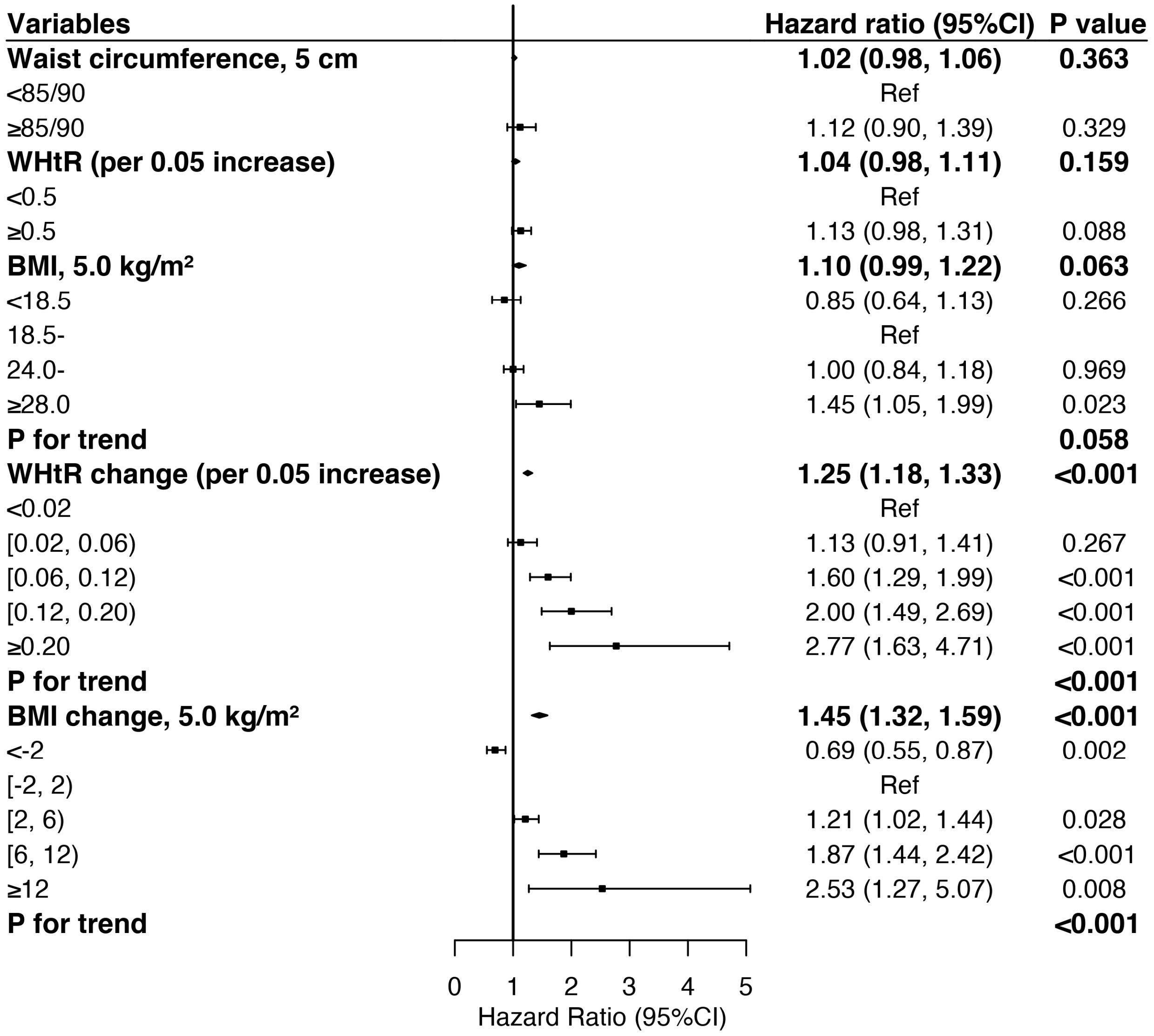

Figure. S3 Sensitivity analysis after excluding participants who died with unclear status of dyslipidemia (2945 remained). HR hazard ratio; 95%CI 95% confidence interval; BMI body mass index; WHtR waist-to-height ratio. **Note:** Adjusted for age (categorical variable), sex, residence, nationality, current alcohol consumption, regular physical exercise, baseline BMI (categorical variable) for variables related to BMI change, and baseline WHtR (categorical variable) for variables related to WHtR change.

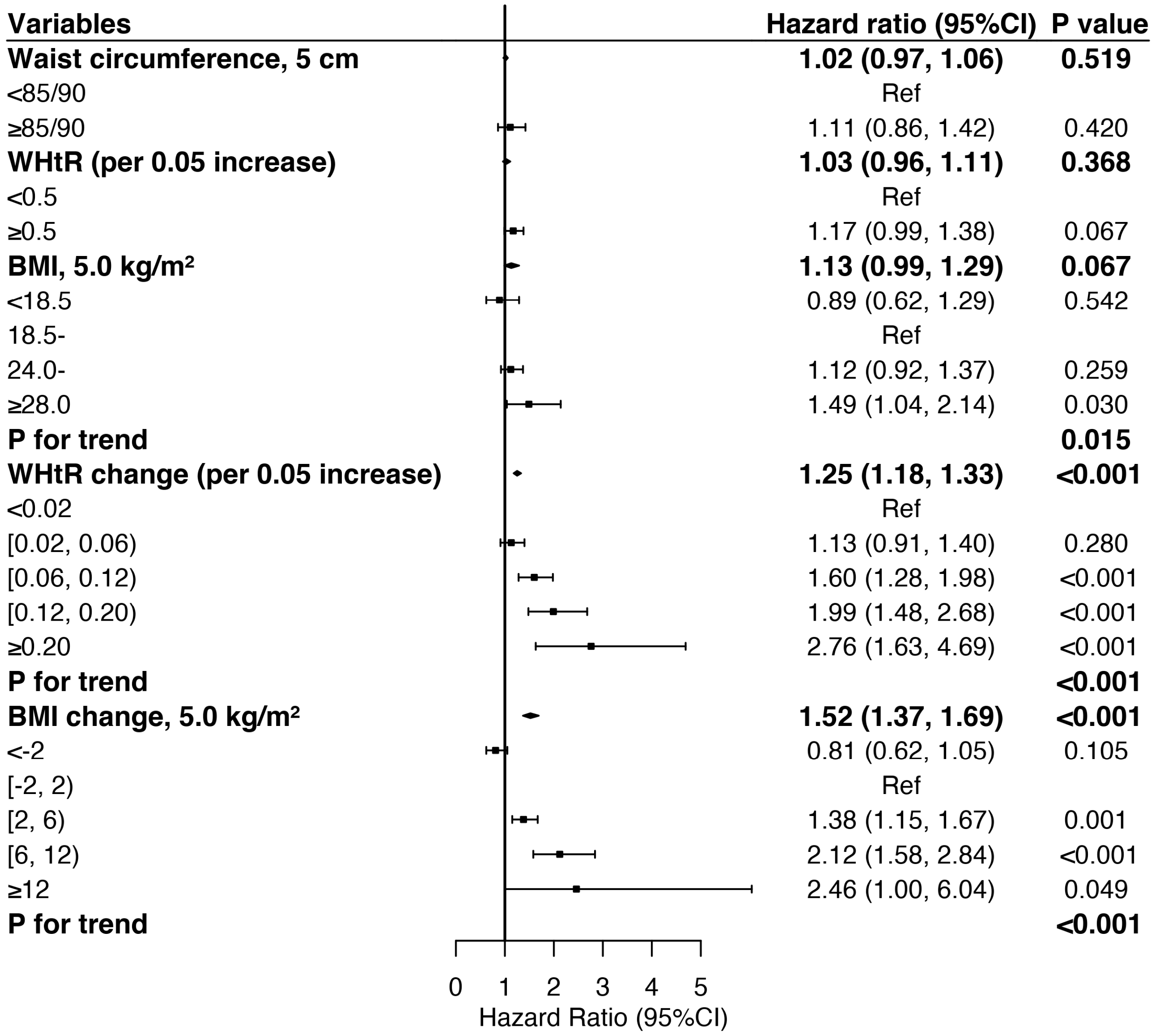

Figure. S4 Sensitivity analysis after excluding participants who has missing values of any covariant (1924 remained). HR hazard ratio; 95%CI 95% confidence interval; BMI body mass index; WHtR waist-to-height ratio. **Note:** Adjusted for age (categorical variable), sex, residence, nationality, current alcohol consumption, regular physical exercise, baseline BMI (categorical variable) for variables related to BMI change, and baseline WHtR (categorical variable) for variables related to WHtR change.
